# Aurantiamide suppresses the activation of NLRP3 inflammasome to improve the cognitive function and central inflammation in mice with Alzheimer's disease

**DOI:** 10.1111/cns.14082

**Published:** 2023-01-10

**Authors:** Heping Shen, Hongyan Pei, Liping Zhai, Qiaobing Guan, Genghuan Wang

**Affiliations:** ^1^ Department of Neurology The Second Affiliated Hospital of Jiaxing University Jiaxing China; ^2^ College of Chinese Medicinal Materials Jilin Agricultural University Changchun China; ^3^ Department of Neurosurgery The Second Affiliated Hospital of Jiaxing University Jiaxing China

**Keywords:** Alzheimer's disease, aurantiamide, inflammatory factors, microglial cells, NLRP3 inflammasome

## Abstract

**Aim:**

This study was aimed at exploring the mechanism by which aurantiamide (Aur) targeted NLRP3 to suppress microglial cell polarization.

**Methods:**

The 7‐month‐old APP/PS1 mice and C57BL/6 mice were applied to be the study objects, and Aur was administered intragastrically to APP/PS1 mice at 10 mg/kg and 20 mg/kg. The changes in the neurocognitive function of mice were measured by Morris Water Maze (MWM) test. In the in vitro experiments, the mouse BV2 cells were employed as the study objects, which were subject to treatment with 10 μM and 20 μM Aur and induced with LPS and IFN‐γ in order to activate BV2 cells and induce their M1 polarization.

**Results:**

Aur was found to suppress the M1 polarization of mouse microglia, reduce central neuroinflammation, and improve the cognitive function in mice. Meanwhile, Aur suppressed the activation and the expression of NLRP3 inflammasome. The results of experiments in vitro demonstrated that Aur inhibited the activation and M1 polarization of BV2 cells.

**Conclusion:**

Aur targets NLRP3 and suppresses the activation of NLRP3 inflammasome.

## Graphical abstract

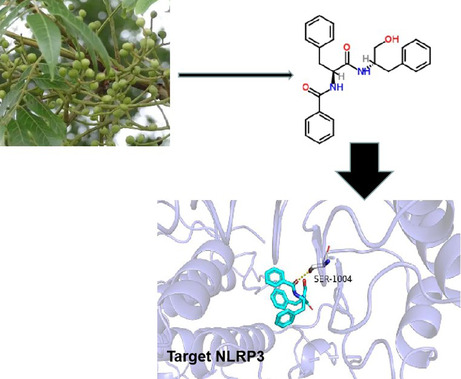

## 1 BACKGROUND

Aurantiamide (Aur) refers to a kind of small‐molecular compound that is extracted from pepper,^1^ which is an important active substance in addition to piperine and pipercide.^2^, ^3^ Currently, there are few reports on the pharmacological activity of Aur, while piperine and pipercide with similar structures have been extensively investigated. Piperine possesses favorable anti‐inflammatory and antitumor activities.^4^ It is shown that piperine exhibits excellent antagonistic effects against experimental electrical stimulation in mice.^5^ Piperine also resists against seizure and audiogenic seizure induced by pentetrazol, picrotoxin, strychnine, intraventricular injection of tubocurarine and glutamic acid to varying degrees.^6^ In addition, it is also effective on certain types of seizure. These neuropharmacological effects are all related to anti‐inflammation. At present, anti‐inflammatory research indicates that Aur can inhibit the virus‐induced inflammatory response by inhibiting the NF‐B signal,^7^ and exerts a good antagonistic effect on influenza A virus infection. Some scholars have also indicated that Aur can generate anti‐inflammatory effects through metabolic signaling and insulin‐like signaling based on network pharmacology research.^8^ However, there is no report on the role and target of Aur in the study of neuroinflammation.

AD studies show that piperine can improve cognitive impairment in AD mice, and its impact is associated with the improvement of synaptic function and the inhibition of inflammation,^9^ while the exact target remains unknown. Considering the similar structure of Aur to piperine, the current work attempted to show the anti‐inflammatory effect and targets of Aur with Alzheimer's disease (AD) as an example.

In the study of AD, it has been discovered that neuroinflammation is an important factor which can stimulate the occurrence and development of AD.^10^, ^11^ The activation and polarization of microglial cells (MG) can be considered to be the main factors leading to neuroinflammation. Based on the existing studies, NLRP3 inflammasome can mediate the activation of MG as well as the release of inflammatory factors.^12^ After the activation, the activated NLRP3 is capable of recruiting the ASC protein to activate the inflammasome, which can further form the protein complex with Caspase‐1. The activation of NLRP3 proves to be a vital hallmark of M1 polarization^13^ and NLRP3 is thus one of the important targets suppressing neuroinflammation. In addition, this study also focused on exploring the association between Aur and NLRP3.

## 2 MATERIALS AND METHODS

### 2.1 Cell culture

After resuscitation, the mouse microglial BV2 cell line (Procell Biotechnology, Co., Ltd) was cultivated in the RPMI‐1640 + 10% FBS complete medium and also subject to incubation based on the conditions of 37°C, 5% CO_2_ and saturated humidity. In order to perform the experiments, the cells at logarithmic phase were harvested. BV2 cells were classified as DMSO, LPS + IFN‐γ (L/I), and Aur groups. LPS + IFN‐γ was regarded as the M1 cell induction group,^14^ where cells were triggered with 200 ng/mL PMA (Sigma) for 6 h. Subsequently, for further induction, 1 μg/mL LPS (Sigma) and 20 ng/mL IFN‐γ (Sigma) were supplemented. In Aur group, BV2 cells were pretreated with 10 μM and 20 μM Aur for 6 h. Thereafter, their M1 polarization was induced based on the same method used in L/I group.

The NLRP3 knockdown cell line (BV2‐*nlrp3* ^*−/−*^, prepared in our laboratory) was adopted in the mechanism research. To be specific, cells were classified as DMSO, L/I, *nlrp3* ^*−/−*^‐L/I and *nlrp3* ^*−/−*^‐L/I + Aur groups. In DMSO group, BV2 cells were used as control, while in L/I group, the M1 polarization of BV2 cells was induced using the above‐mentioned method. As shown in *nlrp3* ^*−/−*^‐L/I group, BV2‐*nlrp3* ^*−/−*^ cells were treated as the objects for M1 polarization induction, while BV2‐*nlrp3* ^*−/−*^ cells in *nlrp3* ^*−/−*^‐L/I + Aur group were subject to pretreatment with 20 μM Aur for 6 h. Thereafter, the M1 polarization was induced.

### 2.2 Flow cytometry (FCM) analysis

To measure the ratio of F4/80+CD11b+M1 cells,^15^ BV2 cells were exposed to an inoculation into the 6‐well plate and M1 polarization was induced after adaptive culture for 12 h. Following LPS/IFN‐γ treatment for 2 days, BV2 cells were gathered, rinsed with pre‐chilled PBS twice, and fixed with methanol. Subsequently, cells were subject to incubation with 10 μl FITC‐F4/80 monoclonal antibody and PE‐CD11b monoclonal antibody (BD) for 20 min in dark. By rinsing twice with PBS, the cells were resuspended with 50 μl of liquid. After the machine detection, the obtained findings could be denoted to be %.

### 2.3 Immunofluorescence staining

In addition, the expressions of CD11b and NLRP3 proteins in BV2 cells were identified. BV2 cells were inoculated on the glass slide to prepare the cell climbing films, treated with LPS and IFN‐γ to cause polarization for 24 h, and then stained. Afterwards, cells were rinsed with pre‐chilled PBS thrice, fixed with pre‐chilled methanol for 0.5 h, as well as permeabilized with 0.2% Triton X‐100 for 5 min. Later, the CD11b and NLRP3 monoclonal antibodies (Abcam, Massachusetts, USA) were diluted with TBST at 1:300 and added to incubate cells at 4°C on the shaking table. By rinsing twice with PBS, cells were subject to incubation with fluorescence secondary antibody, and mounted with 95% glycerin. In addition, we adopted the fluorescence microscope for observation.

### 2.4 ELISA

As shown in BV2 cell experiments, the expressions of M1 cell marker cytokines IL‐1β, IL‐6 and TNF‐α were identified. In FCM, cell medium was gathered after the cell extraction, followed by 30 min centrifugation at 3000 rpm and preservation at −80°C. Then, following the specific instructions, the medium was detected for cytokines with the use of the ELISA kit (Jiancheng Institute of Biology). In addition, the standard curve was used to calculate the expression levels. The results were denoted to be pg/ml.

For animal experiments, the mouse brain tissues were separated, rinsed with PBS twice and grinded until no granule was observed after the removal of tissues including blood vessels and thin membranes. Afterwards, 1 ml NP‐40 lysate (Beyotime Biotechnology Co., Ltd) was supplemented to lyse cells on ice for half an hour. Meanwhile, using the same method in BV2 cell experiments, the supernatant protein solution was harvested for the identification.

### 2.5 ROS detection

The DCFH‐DA probe (Green) and DHE probe (Red) were used to detect reactive oxygen species (ROS). BV2 cells were inoculated into the 6‐well plates and treated with LPS/IFN‐γ for a day. After rinsing with pre‐chilled PBS twice, the DCFH‐DA probe was diluted with serum‐free medium at 1:1000, the DHE probe was diluted at 1:1500, and 1 ml diluted fluorescence probe solution was added into each well to deeply incubate the cells for 30 min. Subsequently, cells were washed twice. Using the fluorescence microscope, the cell staining level was observed. Meanwhile, the fluorescence spectrophotometer was used to detect the absorbance (OD) value.

### 2.6 Western‐blot (WB) assay

As shown in cell experiments, LPS/IFN‐γ were added to induce BV2 cells, followed by rinsing with pre‐chilled PBS twice. After washing twice by PBS, thin membranes and blood vessels were removed from mouse brain tissues, and the brain tissues were grinded until no granule was observed. Later, cells and tissues were digested with 1 ml NP‐40 lysate on ice for 30 min with the purpose of extracting the total proteins. The protein contents were identified. Next, proteins were separated through electrophoresis and transferred onto the PVDF membrane. Then, the PVDF membrane was blocked with 5% defatted milk powder for 2 h. The expression levels of NLRP3, ASC, CD11b and Caspase‐1 were determined. Afterwards, the membrane was subject to incubation with monoclonal antibodies (Abcam) diluted with TBST at a volumetric ratio of 1:300–1:500 at 4°C overnight. Then, the membrane was deeply incubated with HRP‐IgG diluted with TBST at the volumetric ratio of 1:2000. After the incubation, the protein blots were identified with chemiluminescence (ECL), and the OD value was explored by adopting the Image Pro‐Plus 6.0 software. With GAPDH as the internal control, the findings were denoted to be the OD ratio of target protein to internal control protein.

### 2.7 Molecule‐protein docking and pull‐down assays

The NLRP3 receptor protein (PDB ID: 6NPY) was retrieved from the Protein Data Bank database. The box centers (center_x = 88.446, center_y = 95.078, and center_z = 92.124) and the box lattice parameters (size_x = 50, size_y = 60, and size_z = 52) suitable for NLRP3 receptor protein were determined. Afterwards, the active pocket sites possibly bound by the small‐molecular ligand, including NLRP3 receptor protein and Aur ligand small molecule, were subject to molecular docking (AutoDock Vina 1.1.2). Thereafter, the PyMOL was applied to prepare the 3D diagram to display the hydrogen bond interaction between the receptor protein and the ligand small molecule. Meanwhile, the Ligplus software was employed to plot the 2D diagram for displaying the hydrophobic effect between the receptor protein and the ligand small molecule.

Thereafter, 15 μg recombinant NLRP3 protein was bound to the Biotin‐conjugated Aur (Biotin‐Aur). Cells were incubated with the recombinant G protein magnetic bead and NLRP3 antibody. After rinsing with Tris buffer, NLRP3 expression was identified by the aforementioned WB assay.

### 2.8 Mouse grouping experiments

The wild‐type (WT) mice (Normal) and APP/PS1 double‐transgenic AD mice were raided in the Jiaxing University Animal Experimental Center. The mouse experiments were approved by the Ethics Committee of Jiaxing University and performed following the Guides for the Care and Use of Experimental Animals. The 7‐month‐old AD mice presented with senile plaques and symptoms of AD neurological disorder, consistent with the standards of AD research. Mice were classified into WT, AD and Aur groups, with 10 mice (5 females and 5 males) in each group. Mice in Gla group were given intragastric administration of Aur at 10 mg/kg (low‐concentration) and 20 mg/kg (high concentration) once a day for 30 consecutive days.

### 2.9 Morris Water Maze (MWM) test

The MWM and video system were obtained from Feidi Biotechnology Co., Ltd. One day before the experiment, all animals underwent adaptive training. Briefly, they entered through the MWM entrance and swam freely for the 60 s. Mice stood on the platform for the 20 s if they could find it, and later they were returned to their cages. For a 5‐day navigation test, the platform was positioned in the fourth quadrant. Afterwards, we measured the time of mice moving to the entrance and recorded the time spent finding and climbing on the platform. For mice unable to find the platform in the 60 s period, they were trained and stood on the platform for a 20 s period. Escape latency (EL) was regarded as the duration between mice entering the water and searching for the platform. The platform was removed for the spatial probe test. After mice entered the entrance, we noted the frequency crossing the fourth quadrant within a 60 s period as well as retention time on that platform. The mice were tested every 5 days for 30 days consecutively.

### 2.10 Hematoxylin and eosin (H&E) staining

The mouse brain tissues (cerebral cortex) were deparaffinized with xylene, dehydrated with gradient concentrations of methanol (100%, 95% and 80% in succession), rinsed with tap water for 2 min, and stained with hematoxylin for 3 min. After washing with tap water for 2 min, sections were treated with 1% hydrochloric acid alcohol for 2 s, and rinsed by tap water for 2 min. Next, the sections were treated with 1% ammonia water for 20 s and stained with 0.5% eosin alcohol. After gradient alcohol dehydration, the sections were subject to xylene permeabilization and neutral resin mounting. Finally, a microscope was used to observe section pathological changes. Fluorescence staining of tissues.

### 2.11 Fluorescence staining of tissues

CD11b levels within mouse cerebral cortex tissues were examined. Briefly, brain tissues were dehydrated using sucrose solutions (15% and 30%), embedded in OCT, cut into 8‐μm sections using a freezing microtome, and then preserved at −20°C. Sections were washed with PBS, then 5% serum was mixed to block them for 30 min, followed by overnight incubation with CD11b monoclonal antibody under 4°C. Sections were rinsed by PBS three times, with 1‐h incubation using fluorescence antibody in dark. Then, sections were washed with PBS thrice, and mounted by using an anti‐fluorescence quenching agent. Meanwhile, a microscope was used for observation.

### 2.12 Statistical analysis

The measurement data were represented by ($\bar{x}\pm s$). Data were analyzed and processed with SPSS17.0. All data conform to a normal distribution. After homogeneity test of variance, two independent sample t‐test was employed for comparison between two groups, whereas one‐way ANOVA was conducted for comparison among three groups. Two‐way ANOVA was performed for comparison of behavioral test at multiple time points, and the subsequent pairwise comparison between groups was completed by LSD. All the above tests were two‐sided, and the difference of *p* < 0.05 stood for statistical significance.

## 3 RESULTS

### 3.1 Aur inhibited the M1 polarization of BV2 cells

**FIGURE 1:**
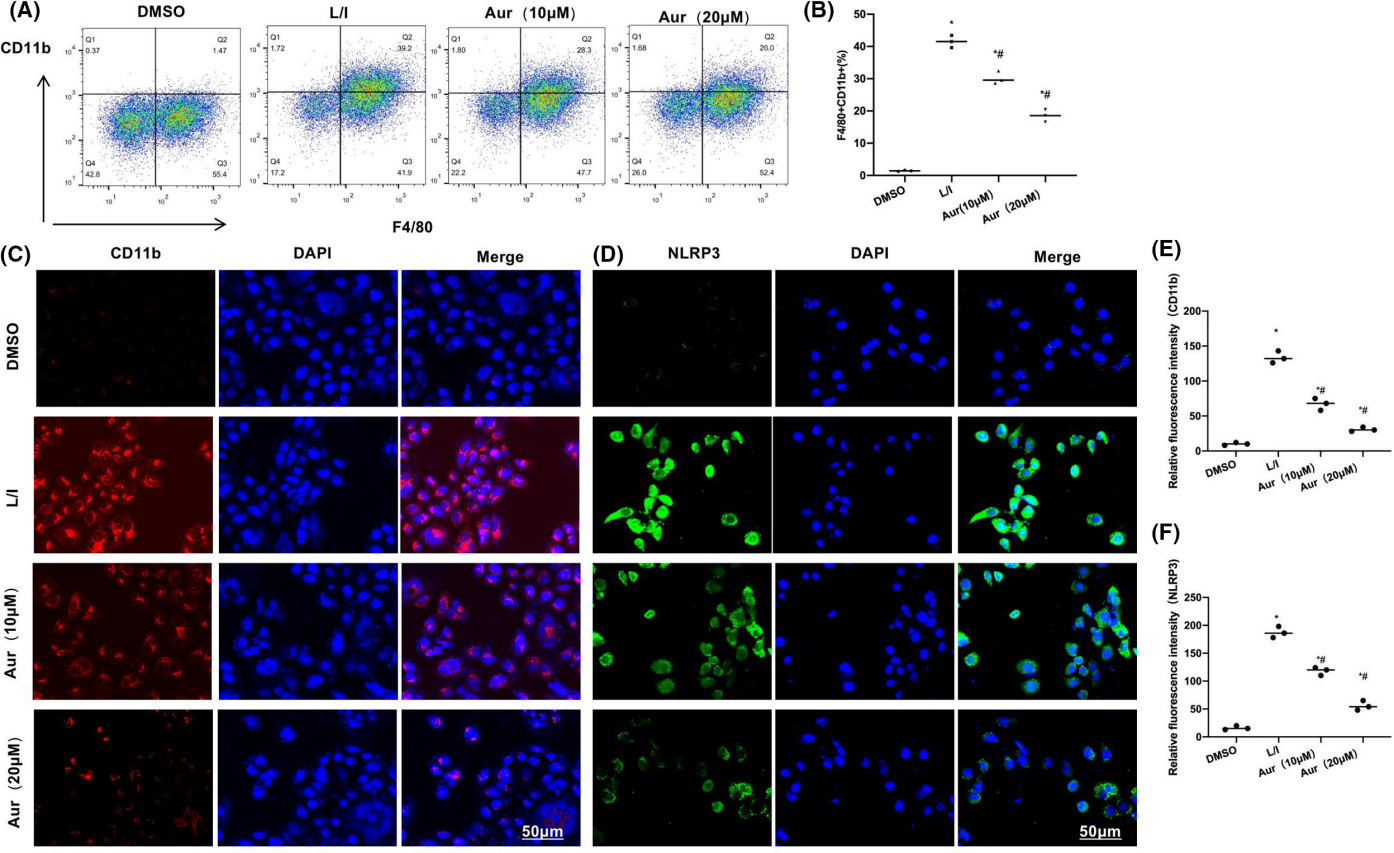
Aur suppresses the M1 polarization of BV2 cells. (A, B) FCM (*n* = 3). Compared with DMSO, the proportion of F4/80+CD11b+ cells in L/I group increased, and Aur hindered the M1 polarization of BV2 cells. Relative to L/I group, the ratio of F4/80+CD11b+ cells obviously lowered, and the high‐dose Aur exhibited better impact than low‐dose Aur. *p < 0.05 in relative to DMSO group, ^#^ *p* < 0.05 in relative to L/I group. B–F: Immunofluorescence staining (*n* = 3). After LPS + IFN‐γ induced the M1 polarization of BV2 cells, the expression of CD11b and NLRP3 notably increased, and the fluorescence intensity was found to be higher when compared with that in DMSO group. Aur inhibited the expression of CD11b and NLPR3 and reduced the fluorescence intensity in a dose‐dependent manner. *p < 0.05 in relative to DMSO group, ^#^ *p* < 0.05 in relative to L/I group.

**FIGURE 2:**
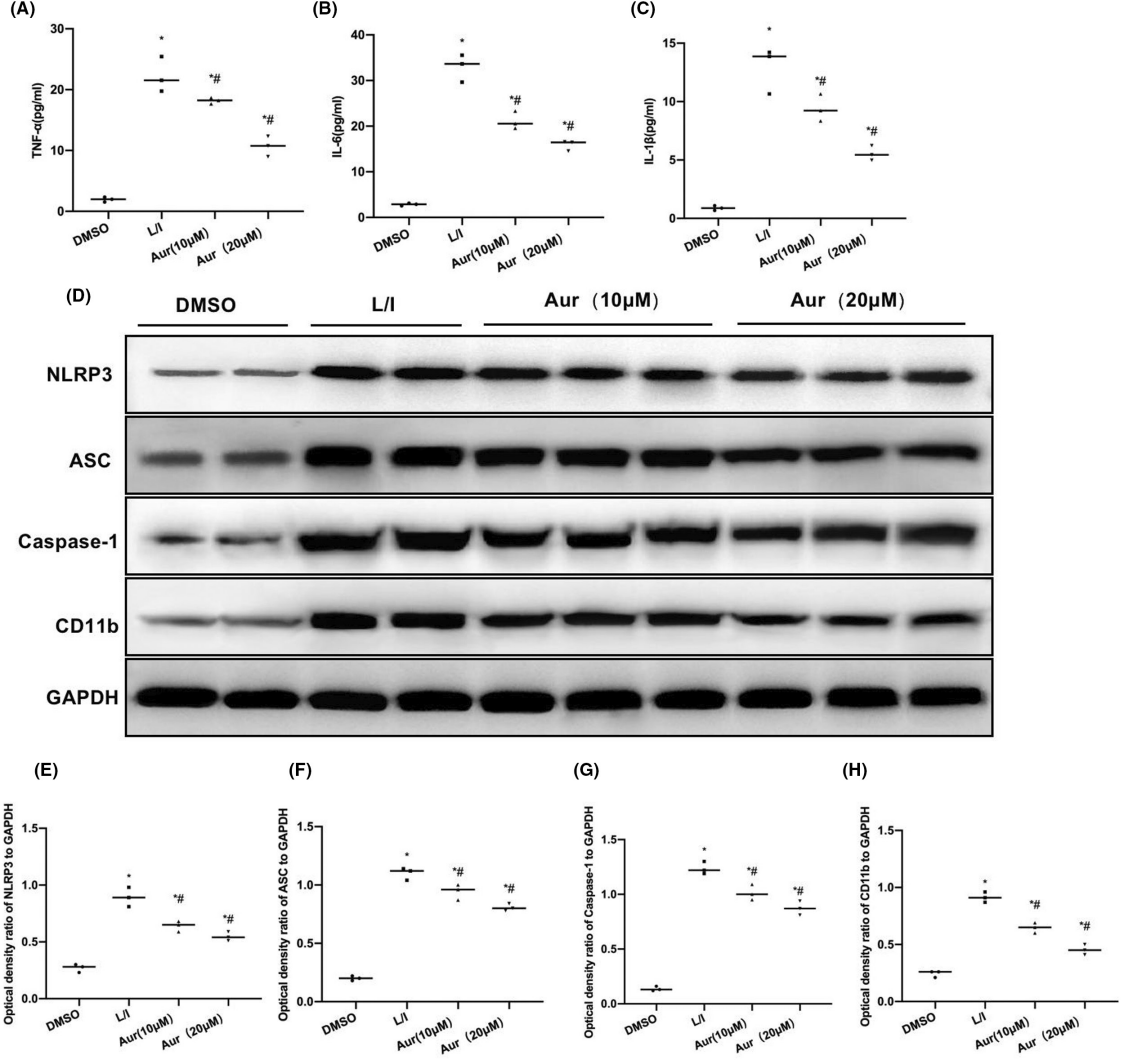
Aur suppresses inflammatory factor expression and NLRP3 inflammasome activation in BV2 cells. (A–C) ELISA (*n* = 3). Detection of inflammatory factors indicated that, the expression levels of inflammatory factors IL‐6, TNF‐α and IL‐1β in DMSO were low, while those in L/I group presented significant upregulation compared with DMSO. The pretreatment of Aur reduced the expression of inflammatory factors in a dose‐dependent manner, and the difference was of statistical significance in comparison with L/I group. *p < 0.05 in relative to DMSO group, ^#^ *p* < 0.05 in relative to L/I group. (D–H) WB assay (*N* = 3). The levels of NLRP3, ASC, Caspase‐1 and CD1b in DMSO were low, while NLRP3 was activated in L/I group, and the protein levels were significantly upregulated, with the significant difference compared with DMSO group. Aur inhibited the activation of NLRP3 inflammasome, reduced protein level in a dose‐dependent manner, and the difference was of statistical significance. *p < 0.05 in comparison with DMSO group, ^#^ *p* < 0.05 in comparison with L/I group.

LPS + IFN‐γ was found to trigger the M1 polarization of BV2 cells, and the proportion of F4/80+CD11b+ cells in L/I group was increased, which was notably higher when compared with that of DMSO group (*p* < 0.05). Aur suppressed the M1 polarization of BV2 cells, and the ratio of F4/80 + CD11b + cells lowered obviously in relative to L/I group (*p* < 0.05). The high‐dose (20 μM) Aur had a superior effect to 10 μM Aur (Figure 1A,B). Results of fluorescence staining suggested that, after LPS + IFN‐γ induced the M1 polarization of BV2 cells, the expression of CD11b and NLRP3 obviously elevated, and that the fluorescence intensity was higher when compared with that of DMSO group. Aur suppressed the expression of CD11b and NLRP3 and reduced the fluorescence intensity in a dose‐dependent manner (Figure 1C–F). According to ELISA results, the expressions of inflammatory factors IL‐6, TNF‐α and IL‐1β in DMSO were low, while those in L/I group significantly increased (*p* < 0.05), consistent with the feature of M1 cells. After the pretreatment of Aur, the expression of inflammatory factors was reduced in a dose‐dependent manner, and the difference was shown to be significant in relative to L/I group (*p* < 0.05) (Figure 2A–C). Based on the detection results of NLRP3 inflammasome‐related protein, the expressions of NLRP3, ASC and Caspase‐1 in DMSO were low, and NLRP3 was not significantly activated. In L/I group, NLRP3 was activated, its protein expression level was significantly elevated, and the difference was obvious in relative to DMSO group (*p* < 0.05). Aur was found to inhibit the activation of NLRP3 inflammasome and reduce the protein levels in a dose‐dependent manner. Additionally, the difference was of statistical significance in relative to L/I group (*p* < 0.05). The CD11b expression level was similar to NLRP3 inflammasome, and Aur inhibited CD11b expression (Figure 2D–H).

### 3.2 Aur inhibited ROS expression and bound to NLRP3

**FIGURE 3:**
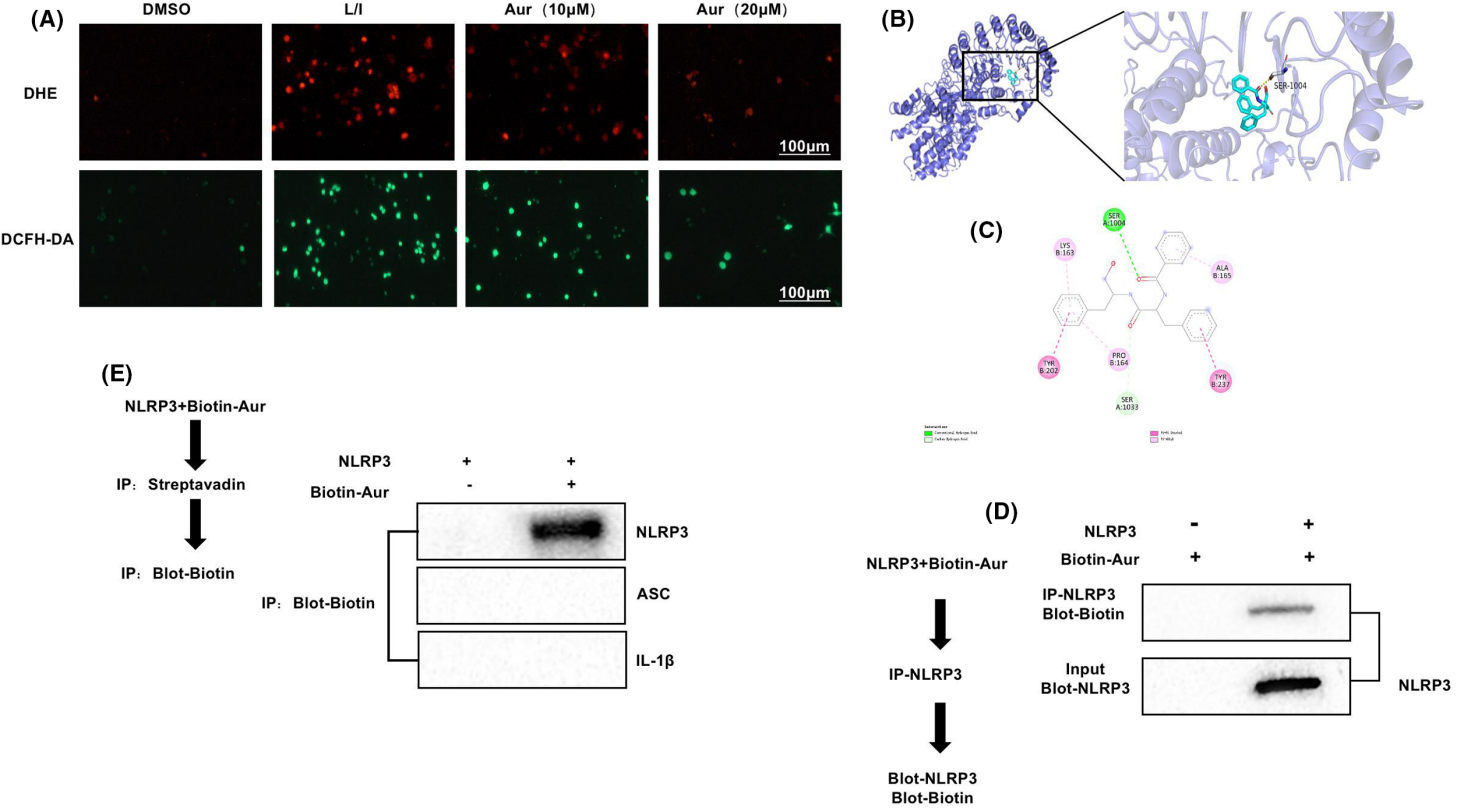
Aur suppresses ROS and specifically binds to NLRP3. (A) DCFH‐DA and DHE (*n* = 3). ROS was not expressed in DMSO, the number of positive cells in L/I group was significantly elevated, ROS expression was upregulated, and the difference was of statistical significance compared with DMSO. The pretreatment of Aur inhibited ROS expression, and the number of positive cells in Aur evidently decreased. (C–E) Docking results of NLRP3 with Aur suggested that there was stable inter‐molecular force between Aur and NLRP3‐SER. Pull‐down assay suggested that Aur bound to NLRP3. Aur bound to SER in the form of hydrogen bonds, and bound to TYR, ALA, LYS, and PRO in the form of hydrophobic bonds.

During the M1 polarization of BV2 cells, ROS was activated. The ROS expression in L/I group was notably upregulated. Both DCFH‐DA and DHE detection results revealed a significantly increased number of positive cells, whereas no positive cell was detected in DMSO. The pretreatment of Aur inhibited ROS expression and decreased the positive cell number (Figure 3A). The docking results of NLRP3 with Aur showed that there was hydrogen bonding between Aur and NLRP3. Meanwhile, Aur bound to SER in the form of hydrogen bonds, and bound to TYR, ALA, LYS, and PRO in the form of hydrophobic bonds. Results of pull‐down assay also indicated that Aur bound to NLRP3 (Figure 3B–E).

### 3.3 NLRP3 knockdown suppressed the effects of Aur

**FIGURE 4:**
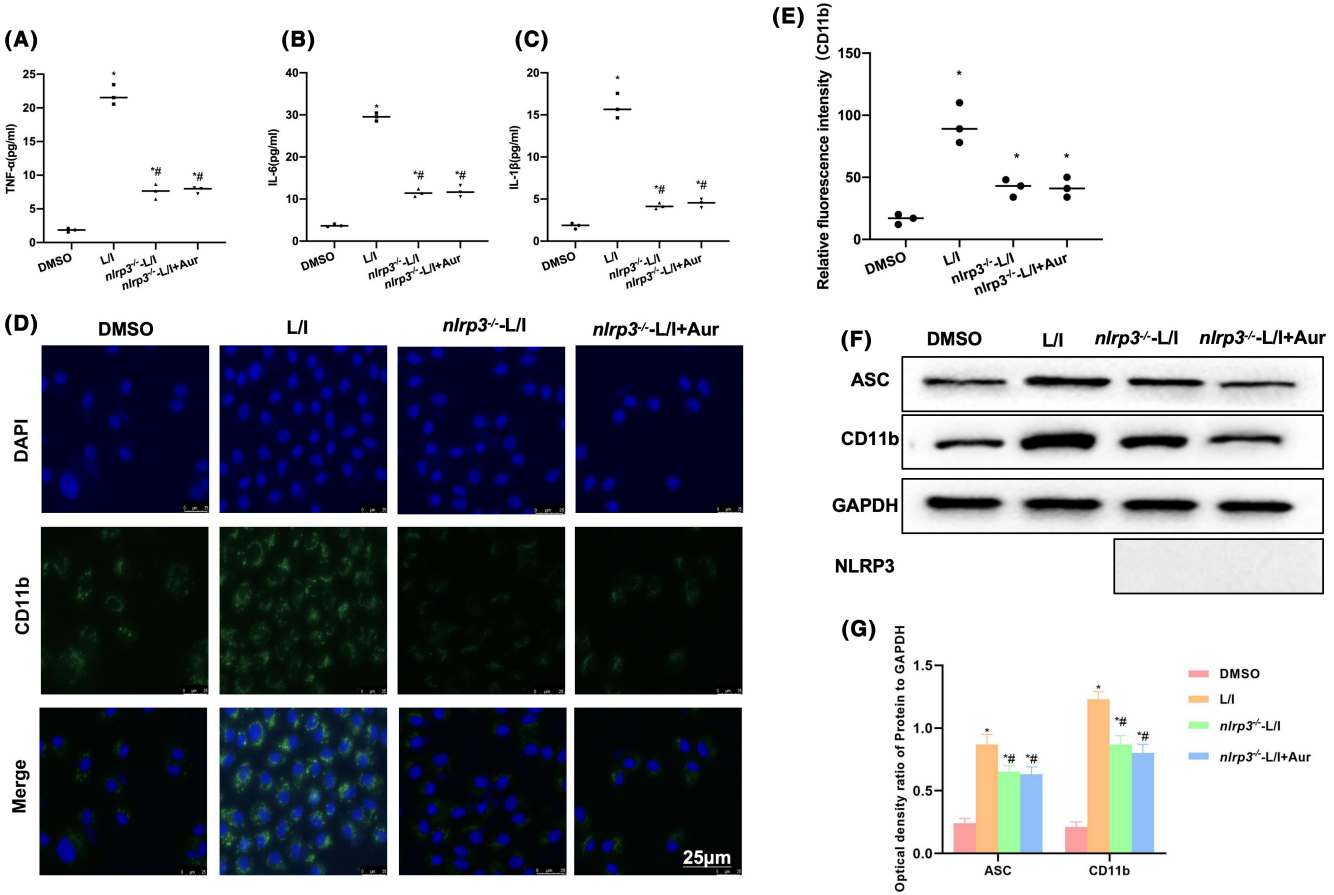
NLRP3 knockdown suppresses the effects of Aur. (A–C) ELISA (*n* = 3). The levels of inflammatory factors in L/I group were significantly higher than those in DMSO group, while those in *nlrp3* ^*−/−*^‐L/I and *nlrp3* ^*−/−*^‐L/I + Aur groups were lower compared with those in L/I group, and the differences were not significantly different. *p < 0.05 compared with DMSO group, ^#^ *p* < 0.05 in relative to L/I group. D, E: Immunofluorescence staining (*n* = 3). After NLRP3 knockdown, CD11b expression was suppressed, and no significant difference was detected between *nlrp3* ^*−/−*^‐L/I and *nlrp3* ^*−/−*^‐L/I + Aur groups. *p < 0.05 compared with DMSO group. F, G: WB assay (*n* = 3). The expression of ASC and CD11b between *nlrp3* ^*−/−*^‐L/I and *nlrp3* ^*−/−*^‐L/I + Aur groups was not obviously different but was significantly lower than that in L/I group. *p < 0.05 in relative to DMSO group, ^#^ *p* < 0.05 in relative to L/I group.

NLRP3 was knocked down in BV2 cells. Inflammatory factor expression findings suggested that there was no significant difference between *nlrp3* ^*−/−*^‐L/I and *nlrp3* ^*−/−*^‐L/I + Aur groups, and that the inflammatory factor levels were significantly lower than those in L/I group (*p* < 0.05). This demonstrated that NLRP3 knockdown decreased the inflammatory factor levels whereas such effect was not related to Aur (Figure 4A–C). Fluorescence staining results indicated that NLRP3 knockdown inhibited CD11b expression, and there existed no significant difference between *nlrp3* ^*−/−*^‐L/I and *nlrp3* ^*−/−*^‐L/I + Aur groups (Figure 4D,E). Protein detection results also revealed no significant difference in ASC and CD11b expression between *nlrp3* ^*−/−*^‐L/I and *nlrp3* ^*−/−*^‐L/I + Aur groups, while their levels were significantly lower than those in L/I group (*p* < 0.05) (Figure 4F,G). According to the obtained results, after NLRP3 knockdown, Aur exerted no obvious influence on the M1 polarization of BV2 cells.

### 3.4 Aur improved the cognitive function of AD mice

**FIGURE 5:**
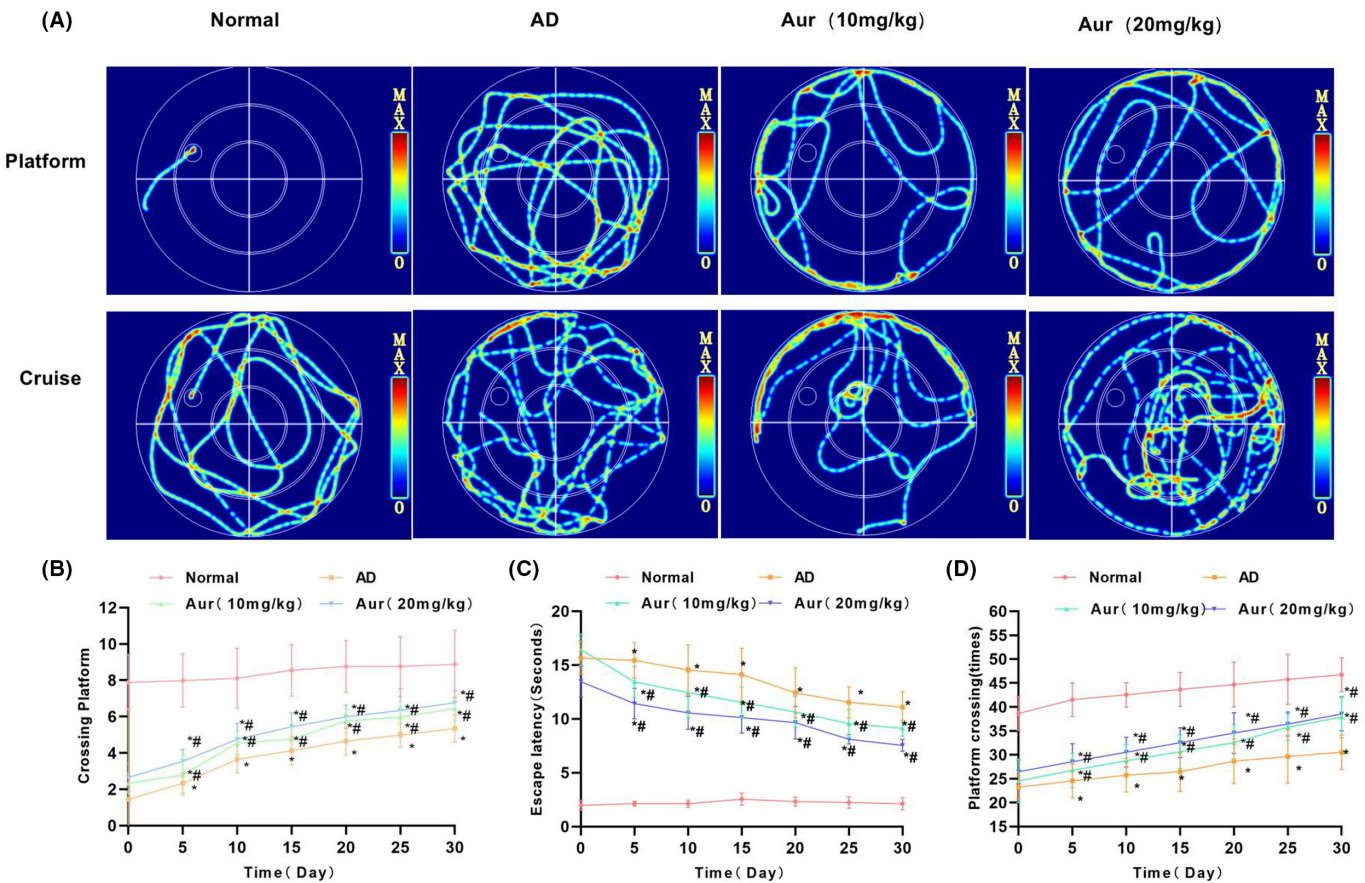
Aur improves the cognitive function in AD mice. (A) Trajectory chart of MWM. (B–D) Cognitive function of mice (*n*=). In comparison with Normal mice, AD mice experienced significant cognitive impairment, less time across the platform, longer EL and longer time to find the platform. Aur could increase the times across platform, reduce the EL, and shorten the time to find the platform. *p < 0.05 in relative to Normal group, ^#^ *p* < 0.05 in relative to AD group.

As presented in the MWM test, AD mice showed significant cognitive impairment in relative to Normal group, with fewer times across the platform, longer EL, and longer time to find the platform, conforming to the cognitive impairment characteristics of AD mice. Aur could improve the times across the platform, reduce the EL, and shorten the time to find the platform. In addition, it also indicated that Aur enhanced the cognitive function of AD mice, and high‐dose Aur exhibited a better impact than low‐dose Aur (Figure 5).

### 3.5 Aur suppressed the polarization of MG and activation of NLRP3 in AD mice

**FIGURE 6:**
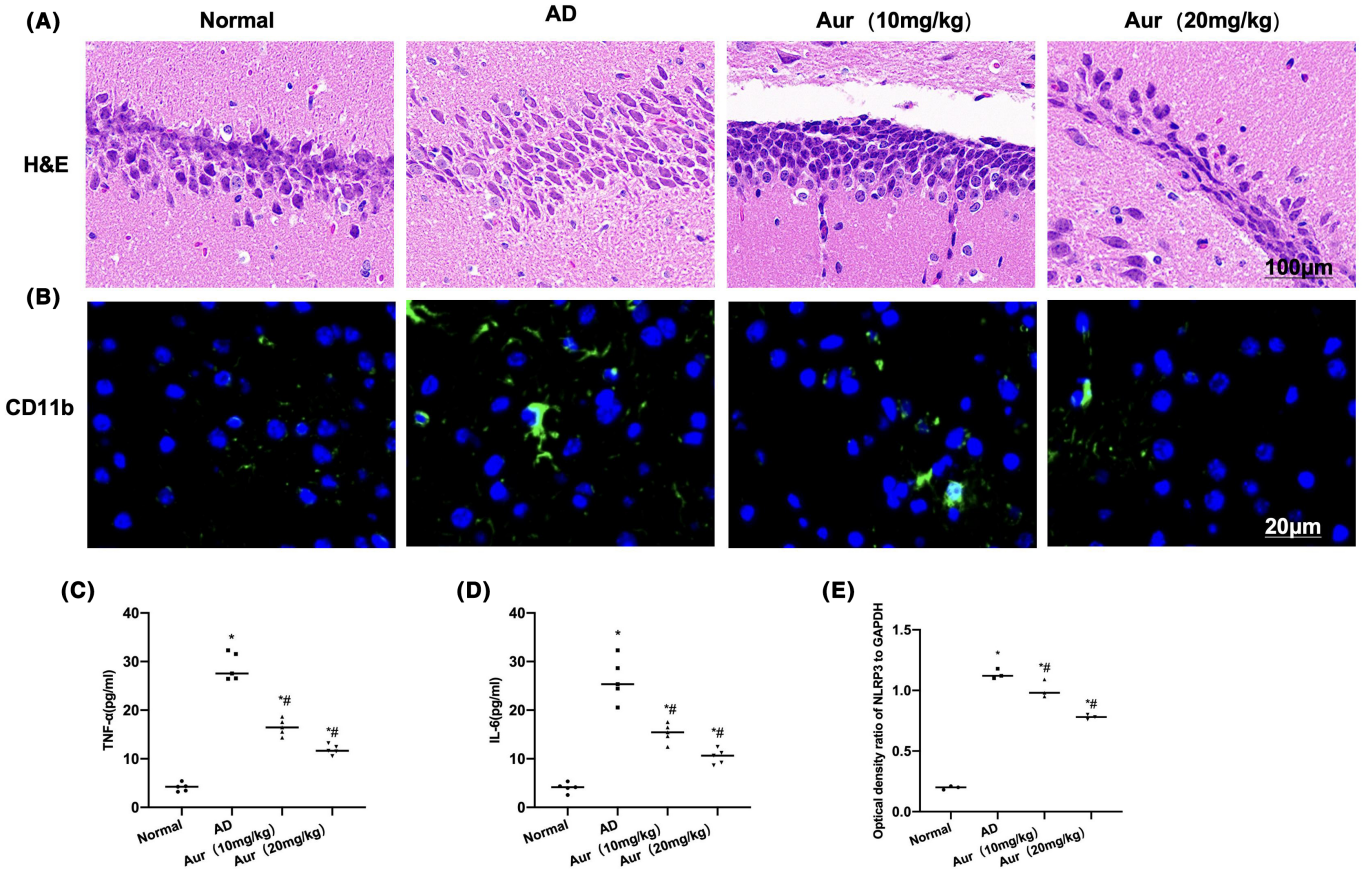
Aur inhibits the activation of MG and the level of inflammatory factors in AD mice. (A) H&E staining (*n* = 5). Normal group showed normal brain tissue morphology, while obvious tissue injury accompanied by inflammatory response was found in AD group, and there existed no obvious difference in comparison with Normal group. In Aur group, the tissue inflammation was obviously alleviated, and the cell injury degree was also mitigated in relative to AD group. (B) Fluorescence staining (*n* = 5). CD11b was not expressed in Normal group but presented significant upregulation in AD group. The difference was of significance in relative to Normal group. Aur inhibited CD11b expression in a dose‐dependent manner. The high‐dose Aur exerted superior effect to low‐dose one. (C–E) ELISA (*n* = 10). The levels of IL‐1β, IL‐6 and TNF‐α in AD group were notably higher than those in Normal group. Aur reduced the inflammatory factor levels in tissues, and the differences were significant in comparison with AD group. Meanwhile, high‐dose Aur exhibited better impact than low‐dose one. *p < 0.05 in relative to Normal group, ^#^ *p* < 0.05 in relative to AD group.

The results of H&E staining indicated no obvious cell injury and inflammatory response in mice of Normal group. In addition, normal brain tissue morphology was found. In AD mice, obvious tissue injury accompanied by inflammatory response was observed. The difference was of significance in relative to Normal group. Tissue inflammation was significantly alleviated in Aur group, and the degree of cell damage is lower than that in AD group, implying that Aur could inhibit AD‐related neural injury (Figure 6A). According to the results of immunofluorescence staining, CD11b was negatively expressed in Normal group, and no obvious polarization of MG was observed, whereas CD11b expression was notably upregulated in AD group, and the difference was significant compared with Normal group. Aur inhibited CD11b expression in a dose‐dependent way (Figure 6B). Based on the detection of inflammatory factors, the expressions of IL‐1β, IL‐6 and TNF‐α in AD were significantly elevated compared with those in Normal group, revealing the presence of obvious inflammatory response in AD. Aur reduced the levels of inflammatory factors in tissues, and significant differences were found compared with AD group. Notably, high‐dose Aur exhibited superior impact to that of low‐dose (Figure 6C–E).

**FIGURE 7:**
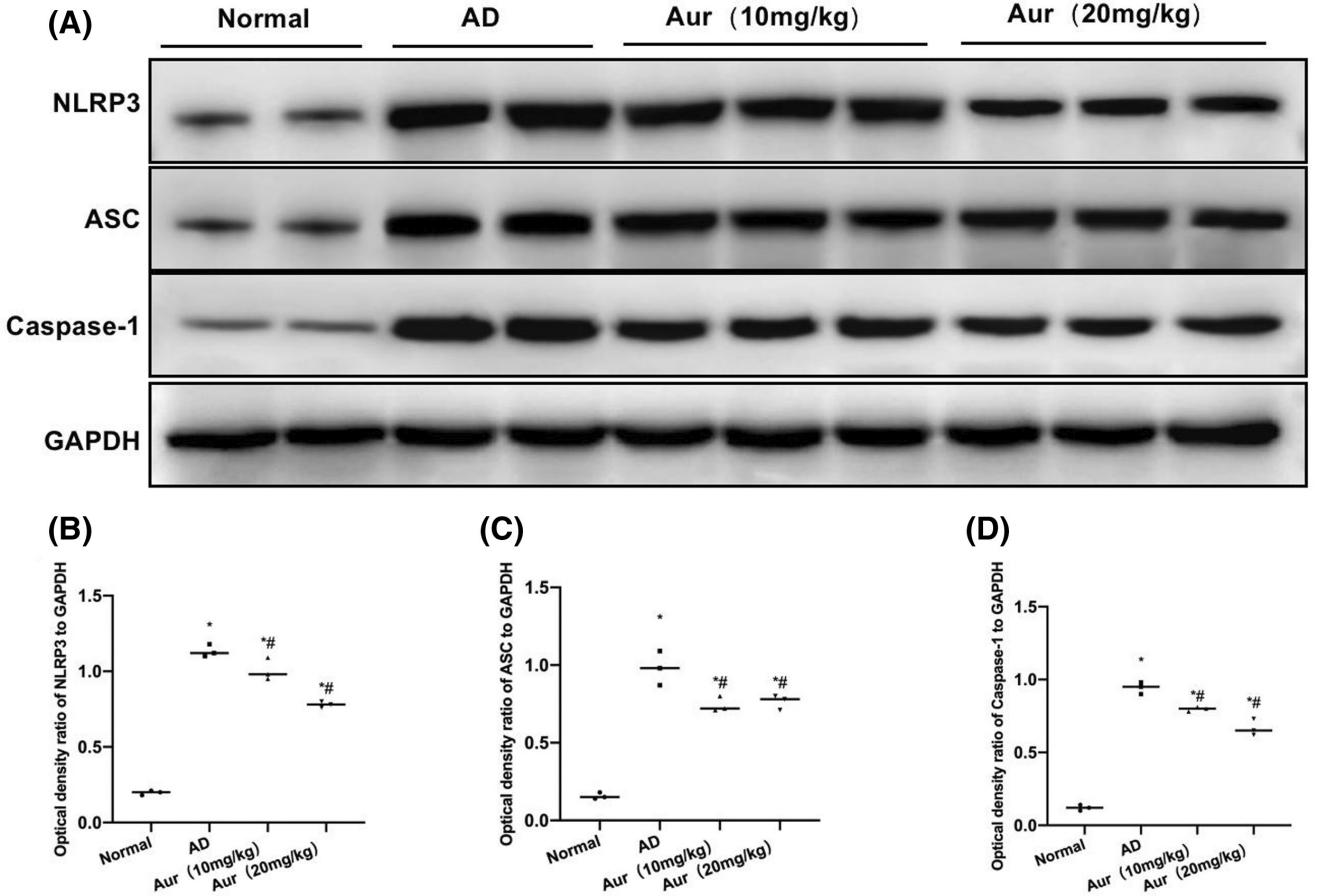
Aur inhibits the activation of NLRP3 inflammasome (*n* = 5). The levels of NLRP3, ASC and Caspase‐1 in Normal group were lower, and NLRP3 was not activated. By contrast, NLRP3 inflammasome and related protein expression levels significantly increased in AD. Aur suppressed the activation of NLRP3, and the expression levels of NLRP3, ASC and Caspase‐1 were notably lower than those in AD group. *p < 0.05 in relative to Normal group, ^#^ *p* < 0.05 in relative to AD group.

The detection of NLRP3 inflammasome indicated that the expression levels of NLRP3, ASC and Caspase‐1 in Normal group were low, and that NLRP3 was not activated. In AD group, the NLRP3 inflammasome and related protein expression levels significantly increased. Aur suppressed the activation of NLRP3, and the levels of NLRP3, ASC and Caspase‐1 were notably downregulated in relative to AD group (*p* < 0.05) (Figure 7).

## 4 DISCUSSION

MG are the immune cells in the central nervous system (CNS), which can regulate the immune homeostasis.^17^ This study suggests that MG can phagocytize and eliminate the abnormal protein aggregates under physiological conditions, which can thus decrease β‐amyloid (Aβ) protein deposition and delay the decline of the cognitive function.^18^, ^19^ However, when MG are abnormally activated, a large number of pro‐inflammatory factors are released, which can induce pro‐inflammatory cascade reaction and lead to neuronal injury.^20^, ^21^ In the healthy brain, MG possesses morphological plasticity and pleiotropy features, and under different activation states, MG can rapidly change the morphology and function in response to the changes in intracerebral microenvironment.^22^ Simulated by multiple factors, MG can transform into the activated M1 and M2 types,^23^ with a larger cell body together with reduced and thickened processes. Different activation phenotypes exert distinct immune effects on the nervous system.^24^ Upon stimulation by immune cells and molecules including LPS, IFN‐γ, β‐amyloid and α‐synuclein,^25^, ^26^ MG are polarized into the classical activated M1 type, which secretes inflammatory mediators including TNF‐α, NO and ROS to damage the peripheral nerve cells.^27^ In neurodegenerative diseases, the abnormal aggregation of Aβ, Tau and α‐synuclein proteins can induce the chronic activation of MG, causing M1/M2 MG cell dysfunction and immunologic inadequacy. Therefore, a large number of inflammatory mediators are released, accelerating Tau protein phosphorylation. The increase in ROS level is also the key factor which can induce the M1 polarization of MG, resulting in the release of pro‐inflammatory cytokines.^28^ The effect of LPS + IFN‐γ is mainly mediated by the Toll‐like receptor (TLR). TLRs can activate inflammasomes, especially NLRP3. NLRP3 inflammasome is formed by NLRP3, ASC and Caspase‐1, exerting its effect by promoting the maturation of pro‐inflammatory factors including pro‐IL‐1β.^29^ NLRP3 is also the key regulatory factor for MG polarization. The disturbance in NLRP3 expression or assembly can suppress the maturation and release of inflammatory factors and inhibit the MG polarization. Therefore, NLPR3 is the vital target for MG polarization and neuroinflammation.

Aur is a derivative of piperine and is also a small‐molecular compound extracted from piperaceae. However, there are currently few studies on the pharmacological effect of Aur whereas piperine, which exhibits a similar structure to Aur, has been extensively investigated. At present, it has been found that piperine exerts vital effects, including anti‐inflammation, antitumor and anti‐bacterium. This study concentrated on the anti‐inflammatory effect of Aur. CD11b is the cell surface antigen of M1 cells, and co‐expresses F4/80‐labeled M1 cells with macrophages. LPS + IFN‐γ treatment is the common method which can be applied to induce M1 polarization. In this study, LPS + IFN‐γ treatment successfully induced M1 polarization of BV2 cells, and upregulated the expression of inflammatory factors, consistent with the phenotypic features of M1 cells. During the polarization process, NLRP3 inflammasome was assembled and activated, and the expression of NLRP3, ASC and Caspase‐1 in the protein complex was also upregulated, indicating that NLRP3 played a vital role in BV2 polarization. After the pretreatment of Aur, the impact of LPS + IFN‐γ was antagonized, and the M1 polarization of BV2 cells was suppressed. In addition, Aur suppressed the maturation and release of inflammatory factors, and also decreased NLRP3 expression. The binding relation between Aur and NLRP3 was confirmed through small molecule‐protein docking and pull‐down assays. Therefore, Aur bound to the SER site, which blocked the assembly of ASC and Caspase‐1 with NLRP3. The blockade of inflammasome assembly further blocked pro‐IL‐β cleavage and polarization, and downregulated CD11b expression. BV2 polarization is mutually promoted by ROS promotes. ROS is excessively expressed during the polarization process, whereas ROS can promote NLRP3 formation and NF‐Κb activation. ROS is one of the important signals that mediate the activation of NLRP3. Two probes were used to detect ROS. The results demonstrated that Aur inhibited the generation of ROS and the activation of NLRP3, and thus ROS and NLRP3 were activated. Our results also suggested that Aur inhibited ROS expression and significantly decreased the positive cell number. To further determine the binding relation between Aur and NLRP3, this study knocked down NLRP3 expression in BV2 cells. Therefore, NLRP3 knockdown antagonized the effect of LPS + IFN‐γ and suppressed the M1 polarization of BV2 cells. After NLRP3 knockdown, Aur lost its activity, which did not further suppress polarization or inhibit the downregulation of inflammatory factors, and it exerted no distinct effect on ASC or NLRP3 expression. As a result, we determined that NLRP3 was the target for Aur.

After the polarization of MG, a large number of inflammatory factors will be released, generating AD progression and aggravation of cognitive impairment,^30^ which are also the important sources of neuroinflammation. In this study, the 7‐month‐old mice with cognitive impairment were detected, which had apparent memory disorder, and the difference was of significance in relative to Normal group. Aur administration could improve the cognitive function, increase the times across the platform, and shorten the time to find the platform, indicating the improved cognitive function of mice. The obtained effect was of significant difference in comparison with AD. Similarly, in AD group, the brain tissue neuroinflammation in mice was improved, the expression of inflammatory factors was downregulated, and cell injury was suppressed. Moreover, these effects were related to the suppression of NLRP3 inflammasome. Experimental results in vitro were consistent with those obtained in vivo. The early efforts to delineate the versatility of microglia/macrophages in stroke brains categorize them into two conceptual phenotypes with pro‐inflammatory (M1) or anti‐inflammatory (M2) functional identities. Although a strict demarcation of M1/M2 polarities is currently known to be oversimplified, the concept of phenotypic diversity is extensively accepted and explored.^31^ Therefore, even though our study finds that Aur can inhibit the polarization of M1 microglia, further exploration is needed for various differential and functional studies. It is also reported that the function of microglia is related to gender differences,^32^, ^33^ Although our study used female and male mice, we did not further evaluate the role of Aur in mice of different sexes. This is also a limitation of our research. In order to further study the role of Aur in microglia of different genders, we need to expand the sample size and conduct research on female and male mice separately to further evaluate the role of Aur。.

## 5 CONCLUSION

Aur can target NLRP3 and suppress its activation, thus regulating the M1 polarization of MG and neuroinflammatory response. Furthermore, Aur can also suppress the cognitive disorder in AD mice, which is a promising small molecule that deserves further investigation.

## AUTHOR CONTRIBUTIONS

Heping Shen and Hongyan Pei are primarily responsible for the operation of the experiment, the acquisition of relevant data and the statistical distraction of the data; Liping Zhai mainly accounts for literature review, project coordination, partial data analysis and article writing; Qiaobing Guan and Genghuan Wang are mainly responsible for project development, financial support, overall project proposal and experimental design.

## FUNDING INFORMATION

This study was funded by the ZheJiang provincial Natural Science Foundation [LGF20C090003] as well as the Science and technology planning project of JiaXing [2020AY30018].

## CONFLICT OF INTEREST

No competing interests.

## CONSENT FOR PUBLICATION

All authors approved the publication of the article.

## Data Availability

The data supporting the findings of the current work can be acquired from the corresponding author upon reasonable request.
