# Corrigendum

**DOI:** 10.1111/cns.14546

**Published:** 2023-11-29

In Shen et al., the author mistakenly inserted the fluorescence results of Figure 4D and Figure 6B during typesetting. The author made modifications and the correct results are as follows:

**Figure:**
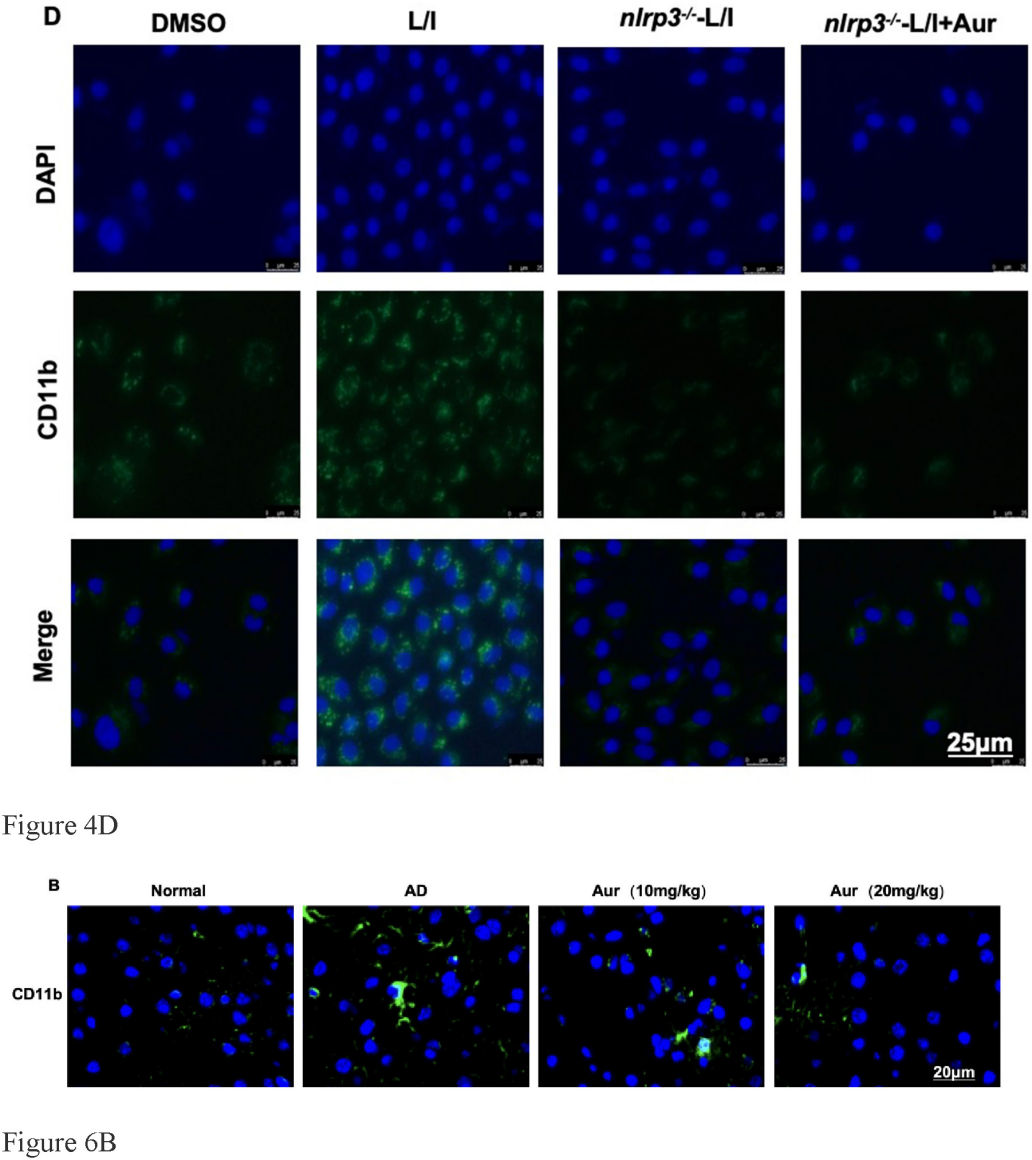
Figure 4D

